# Association between Thyroid Function and Respiratory Distress Syndrome in Preterm Infants

**DOI:** 10.3390/pediatric14040058

**Published:** 2022-11-10

**Authors:** Yonghyuk Kim, Youngjin Kim, Meayoung Chang, Byoungkook Lee

**Affiliations:** 1 Department of Pediatrics, Wonju Severance Christian Hospital, 20, Ilsan-ro, Wonju-si 26426, Korea; 2 Department of Pediatrics, Chungnam National University Hospital, Chungnam National University School of Medicine, 282, Munhwa-ro, Jung-gu, Daejeon 35015, Korea; 3 Department of Pediatrics, Chungnam National University Sejong Hospital, 20, Bodeum 7-ro, Sejong-si 30099, Korea

**Keywords:** respiratory distress syndrome (RDS), thyroid hormone, thyroid-stimulating hormone, preterm infants, thyrotropin

## Abstract

Thyroid hormones are known to influence the production and secretion of pulmonary surfactant. The objective of this study was to explore the relationship between respiratory distress syndrome (RDS) and thyroid hormones. This was a retrospective study of preterm infants at 24–33 weeks gestational age from April 2017 to February 2019. T_3_, free T_4_ (fT_4_), and thyroid-stimulating hormone (TSH) were measured 1, 3, and 6 weeks after birth. Multivariate logistic regression analyses were performed to determine the relationship between RDS and TSH. A total of 146 infants were enrolled. Of these, 60 had RDS, 72 had no RDS, and 14 were excluded. T_3_ and TSH were lower in the RDS groups (*p* < 0.05) on the day of birth. Multivariate logistic regression analysis indicated that lower serum TSH levels immediately after birth were associated with a higher incidence of RDS (OR, 0.89; 95% CI, 0.81–0.97). The TSH level was associated with the incidence of RDS. This suggests that suppression of the hypothalamus–pituitary axis function contributes to RDS, which is the result of surfactant deficiency.

## 1. Introduction

Respiratory distress syndrome (RDS) is a disease caused by inadequate production of pulmonary surfactant and structural immaturity of the lung [1]. Although various treatment modalities are available, RDS is still a major cause of morbidity and mortality in premature infants [1]. The Healthcare Cost and Utilization Project reported an average cost of hospitalization for RDS of 55,000 USD, compared to 3200 USD for all newborns in 2011, with hospital stays of 31.3 days and 3.4 days, respectively [2]. Length of stay and costs for RDS documented in this project were approximately twice those of preterm and low-birthweight (LBW) infants [2]. The average length of stay for newborns who died during hospitalization (including preterm, LBW, and RDS infants) was 7.4 days with an average cost of 286,002 USD. Risk factors affecting the incidence of RDS are known to include prematurity, LBW, maternal diabetes, multiple births, cesarean delivery, precipitous delivery, asphyxia, cold stress, and a maternal history of previously affected infants, among others [3]. Various hormones are known to influence the production and secretion of surfactant, and cortisol is one of them [4,5,6]. In 2006, Gunes et al. reported that lower blood cortisol levels were associated with higher severity of RDS in preterm infants [7]. 

In animal studies, Warburton et al. noted that thyroid hormone promotes fetal lung maturation and has a synergistic effect with glucocorticoids [8]. An antenatal thyrotropin-releasing hormone has been administered to prevent RDS and chronic lung disease in mothers at risk of preterm birth, but no significant results were obtained [9]. Additionally, side effects, including delayed neurodevelopment, were reported in this study [9]. However, Torkaman et al. reported the association of the thyroid-stimulating hormone (TSH) and free T_4_ (fT_4_) with RDS in premature infants [10], and Ryckman et al. reported a strong association between TSH and RDS [11]. In 2007, Tanaka et al. reported that the TSH surge caused by stress during birth improves surfactant production [12]. In this study, fT_4_, triidothyronine (T_3_), and TSH levels were analyzed based on gestational age to assess the relationship between hormone levels and RDS.

## 2. Materials and Methods

This study was a retrospective analysis of premature infants between 24 weeks 0 days and 33 weeks 6 days of gestational age who were born and admitted to the neonatal intensive care unit (NICU) of Wonju Severance Christian Hospital of South Korea between April 2017 and February 2019. Infants with congenital anomalies and congenital hypothyroidism, outborn infants, or those lost to follow-up were excluded. Detailed patient information was extracted from the electronic medical records for analysis. 

The NICU has a protocol for screening preterm infants for thyroid dysfunction. T_3_, fT_4_, and TSH are measured on cord blood on the day of birth and on venous blood 1, 3, and 6 weeks after birth. 

RDS was defined as those infants with air-bronchograms on chest X-ray and respiratory distress symptoms including chest retractions, nasal flaring, and at least a 40% FiO2 requirement. The case group was those infants with RDS, whereas the control group was those without RDS. Bronchopulmonary dysplasia (BPD) was defined as the need for supplementary oxygen at 36 weeks postmenstrual age (PMA) or discharge, whichever came first. Severe BPD was defined as the need for ≥30% oxygen and/or positive pressure at 36 weeks PMA or discharge, whichever came first [13]. Necrotizing enterocolitis (NEC) was defined according to the modified Bell’s criteria, and only stages II or higher were included [14]. Periventricular leukomalacia was defined as the presence of a periventricular cyst on cranial ultrasound or brain magnetic resonance imaging. Mortality was defined as death occurring during the initial admission to the NICU. 

The Statistical Package for the Social Science (SPSS Inc. Chicago, IL, USA) was used for data analysis. Categorical variables were presented as numbers and percentages. Continuous variables such as T_3_, fT_4_, TSH, birth weight, and gestational age were presented as the mean ± SD. Categorical variables were analyzed using the chi-squared test or Fisher’s exact test. Continuous variables were analyzed using the independent t-test, the Mann–Whitney U-test, and repeated measures of ANOVA. The *p*-value for statistical significance was <0.05.

Multivariate logistic regression was performed using stepwise selection to analyze the association between the incidence of RDS and T_3_ and TSH. The 5 min APGAR score, birth weight, and gestational age were adjusted as confounding variables. The results were expressed as odds ratios and 95% confidence intervals. A *p*-value < 0.05 was considered to indicate statistical significance.

## 3. Results

**Figure 1:**
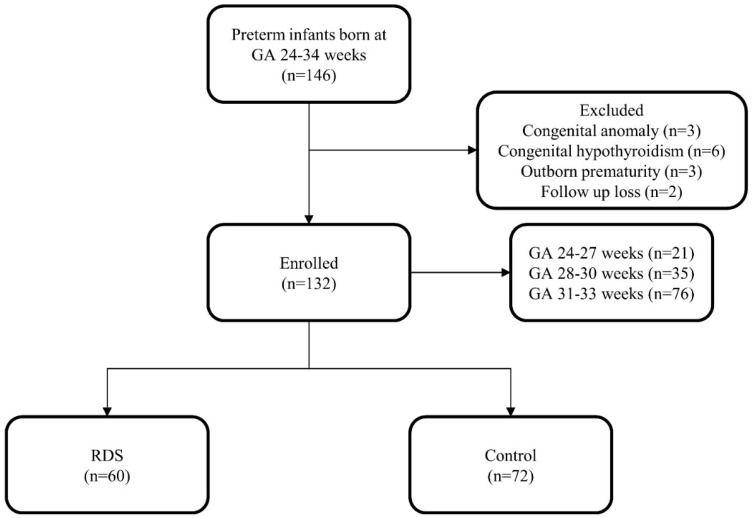
Study population.

Of the 146 premature infants enrolled in the study, 132 were included for analysis. A total of three infants with congenital anomalies, six infants with congenital hypothyroidism, three out-born infants, and two infants who were lost to follow-up were excluded. There were 60 infants with RDS and 72 infants without RDS (Figure 1).

The baseline characteristics of the two groups according to RDS are presented in Table 1. The mean gestational age of the patients was 30.4 ± 2.5 weeks, and the mean birth weight was 1527.2 ± 450.5 g. The mean gestational age and birth weight of the RDS groups were significantly lower than those of the control groups (28.9 ± 2.6 vs. 31.6 ± 1.6, *p* < 0.05; 1328.3 ± 503.1 vs. 1692.9 ± 320.8, *p* < 0.05, respectively). The 5 min APGAR score of the RDS groups was lower than that of the control groups (6.9 ± 1.6 vs. 8.5 ± 1.1, *p* < 0.05), and there were no significant differences in the delivery mode, labor pain, gender, small for gestational age (SGA), maternal age, gestational diabetes mellitus, preeclampsia, chorioamnionitis, or exposure to antenatal steroids. When intergroup comparisons were made based on gestational age, there were no differences in delivery method, gender, multiple births, and SGA, but the 5 min APGAR score tended to be lower for the groups with lower gestational age (*p* < 0.0). 

**Figure 2:**
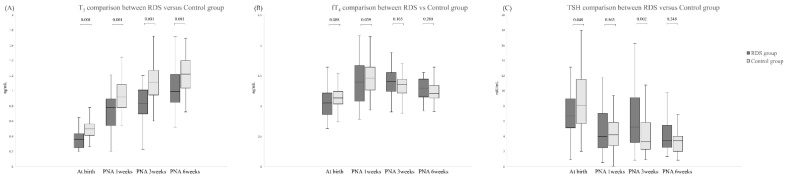
Thyroid hormone levels by postnatal age. (**A**) Triiodothyronine (T_3_) levels increased up to 6 weeks after birth. T_3_ levels were lower in the RDS group on the day of birth and 1, 3, and 6 weeks after birth. (**B**) Free thyroxine (fT_4_) was higher, but not significantly so, in the control group at birth. One week after birth, fT_4_ was significantly lower in the RDS group. (**C**) Thyroid-stimulating hormone levels were higher in the RDS group at birth, but there was no significant difference 1 week after birth.

When serum thyroid hormone levels were compared (Figure 2), both groups had increases in serum T_3_ levels from birth to week 6. However, the T3 levels of RDS groups at every time point were significantly lower (0.39 ± 0.2 vs. 0.49 ± 0.1 at birth; 0.71 ± 0.3 vs. 0.94 ± 0.2 at PNA 1 weeks; 0.83 ± 0.3 vs. 1.10 ± 0.2 at PNA 3 weeks; 1.02 ± 0.3 vs. 1.24 ± 0.3 at PNA 6 weeks; *p* < 0.001). There were no differences between the groups in fT_4_ levels from birth to 6 weeks. TSH levels tended to decrease in both groups following birth. The TSH levels of the RDS groups were significantly lower than that of the control groups at birth (*p* = 0.048), and the TSH level of the RDS groups was increased gradationally. At PNA 3 weeks, levels in the RDS groups were significantly higher than that in the control group (*p* = 0.002). 

Table 2 shows neonatal morbidities for BPD, patent ductus arteriosus requiring treatment, and NEC in the RDS groups compared to those in the control groups (*p* < 0.05). However, no differences were observed for pulmonary hypertension, periventricular leukomalacia, intraventricular hemorrhage, and retinopathy of prematurity requiring panretinal photocoagulation. There was no difference in mortality between the RDS and control groups (*p* = 0.196).

**Figure 3:**
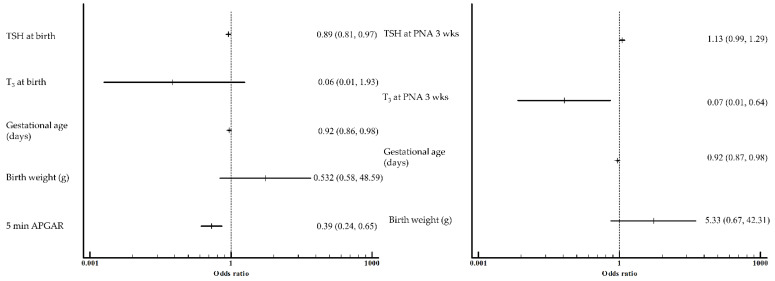
Adjusted odds ratio and their 95% confidence intervals for RDS after multivariate logistic regression analysis adjusted with confounders. Effect of TSH and T_3_ at birth and TSH and T_3_ at PNA 3 weeks. CI, confidence interval; OR, odds ratio; T_3_, triiodothyronine; TSH, thyroid-stimulating hormone; PNA, postnatal age.

On the day of birth, a lower TSH was significantly associated with an increased risk of RDS after adjustment for significant baseline characteristics, including gestational age, birth weight, and 5 min APGAR (Figure 3). At 3 weeks of age, the T_3_ level of the RDS groups was significantly lower than that of the control groups after multivariate logistic regression analysis controlling for gestational age and birth weight. 

## 4. Discussion

This study confirms that serum TSH levels immediately after birth were significantly lower in infants with RDS. Immediately following birth, significant changes occur in the thyroid hormone axis. TSH secretion by the pituitary gland increases up to 70 mIU/L in response to environmental temperature changes within 30 min after birth. This phenomenon is called the TSH surge and stimulates the thyroid to increase serum T_3_ and fT_4_ levels. As a result, serum fT_4_ levels increase to the highest levels in life and then slowly decrease after 1 week [13,14]. The thyroid gland is formed at 7 weeks of gestational age, but T_3_ and fT_4_ secretion does not begin until 12 weeks of gestational age [15]. However, until mid-pregnancy, T_4_ is the primary hormone secreted, and the concentration of T_3_ remains relatively low [16]. Shortly after birth, T_3_ and fT_4_ levels were lower among infants of lower gestational ages. However, as gestational age increased, the significant difference in fT_4_ disappeared over time [16].

Preterm infants have a postnatal TSH surge and rise in serum T_4_ and T_3_, which is qualitatively similar to, but quantitatively smaller than, that of term infants [17,18,19,20]. In this study, all patients had high TSH immediately after birth, and TSH decreased as postnatal maturation progressed. The pattern of TSH surge can be influenced by various factors including lower gestational age, lower body weight, and complications during the fetal period [17,18,19]. Poyekar et al. and Armanian et al. analyzed the effects of gender, perinatal stress, and mode of delivery, and reported that these conditions should be considered in an analysis of TSH levels [19,20]. As noted in previous studies, the TSH surge levels in preterm infants was lower in the RDS groups [18]. 

Previous studies have demonstrated that various hormones such as corticosteroids, thyroid hormones, TRH, prolactin, catecholamines, cAMP, r-interferon, and estrogen affect the surfactant metabolism and lung development in the fetus and newborn [4,5,6]. The thyroid hormone acts on the lungs of the fetus through a receptor-mediated response and increases surfactant production and type II pneumocytes; fT_4_ is thought to promote the translocation of lipids into the lamellar body and increase the influx of phosphatidylcholine precursors to facilitate fetal lung maturation [4], and the potency of T_3_ is much higher than that of fT_4_ [21,22,23]. That provides evidence to show that the T_3_ level at birth in the RDS groups was lower in our study. Additionally, this study also confirmed a significant difference in the TSH level even after adjustment for other confounders, which was different from those in studies of Tanaka et al. [12] or Tawfik et al. [24]. These findings enable the estimating of the correlation between the pituitary–thyroid axis and RDS for a high-rank endocrine system above the thyroid on the axis. However, little is known about the association between RDS and the hypothalamic–pituitary–thyroid axis. Ataoglu et al. analyzed the association between transient tachypnea of the newborn and TSH levels through a cross-sectional study but did not obtain any significant results [25]. Tawfik et al. reported a partial association between RDS and TSH that was similar to that in the results of the present study [24]. Tawfik’s study could not confirm the association between RDS and TSH after adjustment for infant weight; however, this study confirmed that lower TSH at birth was associated with RDS even after adjustment for multiple factors including birth weight. To the best of our knowledge, this is the first report confirming the association between TSH and RDS. Based on prior studies, it was thought that TSH was reduced by pituitary gland suppression, rather than through a direct association with thyroid hormone, which induced the downregulation of thyroid transcription factor-1 in type II cells in alveolus, thereby interfering with surfactant metabolism. 

The hypothesis of this study is supported by the ACTOBAT study, which reported in 1995 that prenatal TRH administration could increase RDS [9]. However, it should be noted that artificial interventions on the hypothalamic–pituitary axis can have multiple adverse effects on the developing organs of preterm infants, since hormones have systemic effects [26]. In 1998, studies attempting to prevent RDS by administering TRH were unsuccessful, and the infants given TRH experienced adverse consequences such as neurodevelopmental delay [26,27]. Contrary to expectations, it was presumed that the effect of hypothalamic suppression by negative feedback on the hypothalamic–pituitary–thyroid axis may have had an adverse effect on neurological development [9,26,27]. The association between RDS and hormones other than thyroid hormone is also being studied. It is well known that the hypothalamic–pituitary–adrenal axis in the fetus affects lung surfactant metabolism [5]. Chu and Rooney confirmed that estrogen stimulates the synthesis and production of lung surfactants [28], and Engle et al. reported that low-dose insulin increased the synthesis of lung surfactants [29]. Multiple hormones are involved in surfactant metabolism and lung development suggesting that hypothalamic function may play an important role. Even in premature infants, the function of the pituitary axis remains intact. The authors suggest that the lowering of terminal hormone, such as cortisol, and thyroid hormone resulted from the suppression of the upper rank in endocrine system of various hormonal axes is related to RDS. 

In the present study, TSH tended to decrease after the initial surge following birth, whereas T_3_ and fT_4_ increased after birth. At 3 weeks of age, TSH appeared to be higher in the RDS groups, but there was no difference after adjustment with gestational age and birth weight. Additionally, this inexistence of difference may be related to the normalization of pituitary gland function by 3 weeks of age or the timing of discontinuation of temperature control of the incubator.

The strength of this study is that it is the first report of an association between TSH and RDS after confounding factors have been eliminated and consistency of care by a single neonatologist in a single center. However, because this is a single-center study, the small sample size is a limitation, and additional studies with a larger number of infants are warranted. 

In this study, lower TSH levels immediately after birth were associated with an increased risk of RDS, but T_3_ and fT_4_ were not associated with RDS. Surfactant deficiency, the cause of RDS, was not affected by thyroid hormone but was associated with suppression of pituitary gland function at birth. Based on these findings, additional research is required to identify the specific endocrinological function that is related to surfactant deficiency.

## Figures and Tables

**Table 1:** Demographics and clinical data.

Variables	Total (*n* = 132)	RDS (*n* = 60)	Control (*n* = 72)	*p* Value
Gestational age (weeks)	30.4 ± 2.5	28.9 ± 2.6	31.6 ± 1.6	<0.05
Birth weight (gram)	1527.2 ± 450.5	1328.3 ± 503.1	1692.9 ± 320.8	<0.05
C-section delivery (%)	102 (77.3)	49 (81.7)	53 (73.6)	0.303
Labor pain (%)	93 (73.8)	43 (71.7)	54 (75)	0.696
Male (%)	72 (54.5)	33 (55.0)	39 (54.2)	1
Multiple gestation (%)	31 (23)	11 (18.3)	20 (27.8)	0.055
Small for gestational age (%)	4 (3)	1 (1.7)	3 (4.2)	0.63
APGAR at 5 min	7.8 ± 1.6	6.9 ± 1.6	8.5 ± 1.1	<0.05
Maternal age (years)	32.7 ± 5.7	32.6 ± 6.7	32.8 ± 4.8	0.789
GDM (%)	19 (14.4)	7 (11.7)	12 (16.7)	0.464
Preeclampsia (%)	29 (22)	16 (26.7)	13 (18.1)	0.292
Pathological chorioamnionitis (%)	53 (44.9)	20 (37)	33 (51.6)	0.139
Maternal thyroid disease (%)	6 (4.5)	4 (6.7)	2 (2.8)	0.41
Antenatal steroids (%)	69 (52.7)	33 (55.5)	36 (50.7)	0.296

Number (%) or mean ± SD shown GDM, gestational diabetes mellitus; PPROM, preterm premature rupture of membrane; SD, standard deviation; SGA, small for gestational age.

**Table 2:** Morbidity and mortality related to RDS.

Variables	RDS (*n* = 60)	Control (*n* = 72)	*p* Value
BPD	28 (50.0)	6 (8.6)	<0.001
PDA (Treated)	15 (26.8)	2 (2.9)	<0.001
Pulmonary hypertension	5 (8.9)	1 (1.4)	0.061
NEC	9 (16.1)	1 (1.4)	0.030
PVL	1 (1.8)	1 (1.4)	0.693
IVH	0 (0.0)	1 (1.4)	0.556
ROP with PRP	5 (8.9)	1 (1.4)	0.061
Death	2 (3.6)	0 (0.0)	0.196

BPD, bronchopulmonary dysplasia; IVH, intraventricular hemorrhage; NEC, necrotizing enterocolitis; PDA, patent ductus arteriosus; PVL, periventricular leukomalacia; RDS, respiratory distress syndrome; ROP with PRP, retinopathy of prematurity with panretinal photocoagulation.

## Data Availability

Data of the present study can be made applicable upon reasonable request to the corresponding author (B.L.).
